# Monitoring CD27 Expression to Evaluate Mycobacterium Tuberculosis Activity in HIV-1 Infected Individuals In Vivo

**DOI:** 10.1371/journal.pone.0027284

**Published:** 2011-11-07

**Authors:** Alexandra Schuetz, Antelmo Haule, Klaus Reither, Njabulo Ngwenyama, Andrea Rachow, Andreas Meyerhans, Leonard Maboko, Richard A. Koup, Michael Hoelscher, Christof Geldmacher

**Affiliations:** 1 NIMR-Mbeya Medical Research Programme, Referral Hospital, Mbeya, Tanzania; 2 Department of Infectious Diseases and Tropical Medicine, Klinikum of University of Munich, Munich, Germany; 3 Immunology Laboratory, Vaccine Research Center, National Institute of Allergy and Infectious Diseases, National Institutes of Health, Bethesda, Maryland, United States of America; 4 Institute of Infections Medicine, Institute of Virology, Saarland University, Homburg/Saar, Germany; University of Stellenbosch, South Africa

## Abstract

The level of bacterial activity is only poorly defined during asymptomatic *Mycobacterium tuberculosis* (MTB) infection. The objective was to study the capacity of a new biomarker, the expression of the T cell maturation marker CD27 on MTB-specific CD4 T cells, to identify active tuberculosis (TB) disease in subjects from a MTB and HIV endemic region. The frequency and CD27 expression of circulating MTB-specific CD4 T cells was determined in 96 study participants after stimulation with purified protein derivative (PPD) using intracellular cytokine staining for IFNgamma (IFNγ). Subjects were then stratified by their TB and HIV status. Within PPD responders, a CD27^−^ phenotype was associated with active TB in HIV^−^ (p = 0.0003) and HIV^+^ (p = 0.057) subjects, respectively. In addition, loss of CD27 expression preceded development of active TB in one HIV seroconverter. Interestingly, in contrast to HIV^−^ subjects, MTB-specific CD4 T cell populations from HIV^+^ TB-asymptomatic subjects were often dominated by CD27^−^ cells. These data indicate that down-regulation of CD27 on MTB-specific CD4 T cell could be used as a biomarker of active TB, potentially preceding clinical TB disease. Furthermore, these data are consistent with the hypothesis that late, chronic HIV infection is frequently associated with increased mycobacterial activity in vivo. The analysis of T cell maturation and activation markers might thus be a useful tool to monitor TB disease progression.

## Introduction

Tuberculosis (TB) is amongst the most frequent causes of death from infection in humans, accounting for an estimated 1.8 million deaths annually (WHO TB Factsheet, 2009) and frequently affects immunocompromised patients co-infected with the human immunodeficiency virus-1 (HIV). In MTB endemic regions, up to 50% of HIV-infected individuals will eventually develop active TB in the absence of antiretroviral therapy [1]. In HIV-infected subjects, TB progresses rapidly and is characterized by a high mortality if left untreated [2]. It is commonly assumed that in HIV-infected individuals active TB is frequently caused by reactivation of a latent MTB infection with an increased MTB activity during chronic HIV-infection.

Diagnosis of active TB is usually based on characteristic X-ray findings, clinical symptoms, tuberculin skin test (TST) and positive sputum-smear microscopy or culture results. In HIV-infected patients TB diagnosis is hampered by lower bacillary load in sputum [3] during pulmonary TB and reduced delayed type hypersensitive reactions during the TST [4]. Interferon-gamma (IFNγ) release assays (IGRAs), which detect and quantify MTB-specific cellular immune responses can improve diagnosis of MTB infection [5]. However, IGRAs do not discriminate between “latent” MTB infections (LTBI) and active TB. Furthermore, the clinical term “latency” might indeed cover a spectrum of manifestations ranging from complete elimination of MTB to continuous low level replication in the absence of clinical TB disease [6]. Hence, there is no reliable test to rapidly diagnose and monitor MTB activity in patients at high risk of developing clinical disease such as HIV-infected patients or children.

MTB is largely controlled by cell-mediated immune responses. Particularly CD4 T cells, which are gradually lost during HIV infection, are thought to play a central role in controlling MTB infection [7], and indeed MTB-specific CD4 T cells are depleted relatively early in subjects who become HIV infected [8]. Maturation of pathogen-specific CD4 T cells is associated with changes in the expression pattern of cell surface proteins, such as CD27. CD27, a receptor involved in co-stimulation, is expressed on the surface of CD4 T cells and is down regulated with advancing cell differentiation from early differentiated CD27^+^ memory T cells to late differentiated CD27^−^ memory T cells. Early differentiated CD27^+^ memory CD4 T cells are thought to mainly re-circulate within the secondary lymphoid organs, whereas CD27^−^ late differentiated memory T cells exhibit additional effector functions and preferentially migrate into peripheral sites of inflammation, such as the lung during active TB disease [9].

Recently published data suggests that flow-cytometric analysis of CD27 expression on circulating MTB-specific T cells can help to discriminate active TB from LTBI [10]. The present study expands upon these findings and investigates CD27 expression on MTB-specific CD4 T cell in relation to HIV and TB status within a large cohort from Tanzania, a MTB and HIV high endemic region. Our results indicated that monitoring CD27 expression on MTB-specific CD4 T cells could be used as a biomarker of active TB in HIV^−^ and HIV^+^ subjects, potentially proceeding active TB. Furthermore the results support the hypothesis that late, chronic HIV infection is frequently associated with increased mycobacterial activity even in TB asymptomatic subjects.

## Materials and Methods

### Study subjects

A cross-sectional analysis of CD27 expression on PPD-specific CD4 T cells was conducted in a cohort of 96 study participants from the Mbeya Region, Southwest Tanzania. The study was approved by the Mbeya Ethics and Research Committee, Tanzania, and the National Ethical Committee/Medical Research Coordinating Committee, National Institute for Medical Research, Tanzania. All participants gave written informed consent. The examination for TB disease included clinical assessment, chest X-ray, white blood cell count, 2× TB staining (Ziehl-Neelsen) of sputum, and 2× TB liquid culture (MGIT). Active TB or symptomatic TB was defined by the presence of acid-fast bacilli in at least one out of two smear sputum samples after Ziehl-Neelsen staining and identification of MTB in BACTEC MGIT 960 (Becton Dickinson, Sparks, USA) liquid culture isolates using the GenoType® test system (Hain Lifescience, Nehren, Germany). Chest X-ray was routinely done in all TB suspects. Participants were classified as TB asymptomatic (TB^−^) when they did not show any symptoms suggestive of pulmonary TB and when no acid-fast bacilli were detected in two sputum samples by microscopy and liquid culture. Symptoms suggesting active TB were defined as persistent cough for more than 2 weeks, haemoptysis, chest pain, fever, night sweats, malaise, recent unexplained weight loss and loss of appetite.

HIV-1 status was determined using repeated positive results on enzyme immunoassay and Western blotting. Subsequently participants were divided into 4 groups according to their TB and HIV status (Table 1). HIV^−^ (n = 10) and HIV^+^ antiretroviral therapy (ART) naive patients (n = 18) with confirmed active TB (active TB), and HIV^−^ (n = 32) and HIV^+^ (n = 36) volunteers without any symptoms suggestive of active TB disease (TB^−^) were included in this study. 26 subjects had a viral load <400 RNA copies/ml. HIV^+^/TB^−^ volunteers were recruited at the Mbeya Referral Hospital HIV/AIDS care and treatment clinics and included 25 subjects on ART. Patients with active TB disease were recruited in collaboration between the Mbeya Regional TB and Leprosy Programme and the Mbeya Medical Research Programme (MMRP) and subsequently received TB treatment. Additionally two HIV seroconverters with positive PPD-responses from the previously described HISIS cohort were included for longitudinal analysis of CD27 expression [11].

**Table 1:** Group characteristics and immunological results of the 96 subjects enrolled in the cross-sectional study.

	N	ART	Previous TB self-reported	PPD responders	PPD^+^ CD27 Ratio <1	Median CD27 Ratio	Diagnostic Concordance MTB Culture*
TB^−^/HIV^−^	32	NA	1	19 (59%)	4	1.42	79%
TB^+^/HIV^−^	10	NA	0	8 (80%)	8	0.48	80%
TB^−^/HIV^+^	35	25	9	14 (40%)	9	0.92	75%
TB^+^/HIV^+^	18	0	0	16 (89%)	14	0.27	78%

NA = not applicable; ART = antiretroviral treatment; PPD^+^ CD27 Ratio = number of PPD-specific CD4 T cells positive for IFN gamma divided by the number of respective CD27^−^ CD4 T cells.

*Diagnostic concordance MTB culture defines the proportion of concordant results defined by PPD response and CD27 expression of PPD-specific CD4 T cells.

### Flow Cytometry

**Figure 1:**
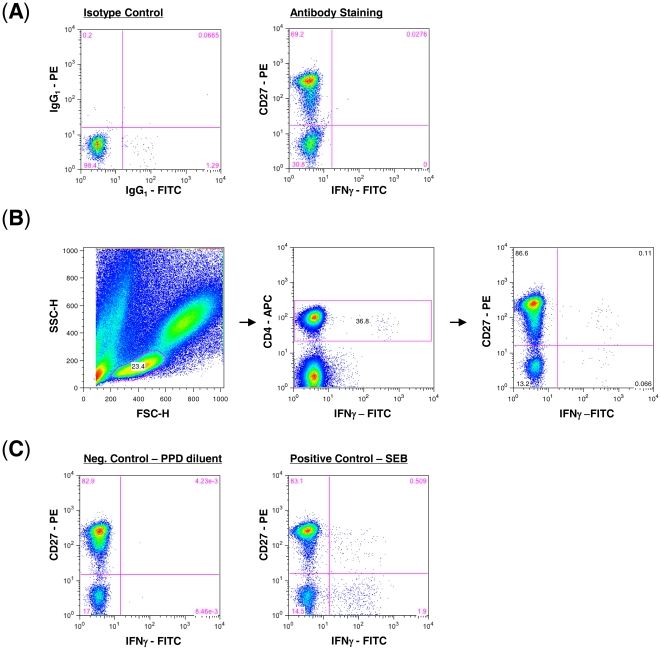
Characterization of CD27 expression on PPD-specific CD4 T cells in whole blood. (A) The cut off for CD27 expression was determined based on the corresponding isotype control for each antibody. Shown is a representative staining for IFNγ-FITC and CD27-PE as well as their corresponding IgG_1_ isotype controls that were used to determine the cut off. (B) Gating strategy to identify PPD-specific CD4 T cells and to analyse the CD27 expression on IFNγ-positive CD4 T cells after 6 h of PPD-stimulation during the cross-sectional study. (C) Representative staining for negative and positive controls using PPD diluent and SEB, respectively, for stimulation.

For the cross-sectional study, the frequency and phenotype of MTB-specific CD4 T cells was determined by intracellular cytokine staining for IFNγ after 6 h stimulation of fresh whole blood with 10 µmg/ml Purified Protein Derivative (PPD, Serum Staten Institute) at 37°C and 5% CO2 as previously described [12]. The following antibodies were used CD4-allophycocyanin (APC), IFNγ-fluorescein isothiocyanate (FITC) and CD27-phycoerythrin (PE, all Becton Dickinson). At least 30,000 cells were acquired and analyzed on a FACS Calibur (Becton Dickinson). The cut off for each antibody was determined based on the corresponding isotype control. The following corresponding isotype controls were used IgG_1_-APC (mouse), IgG_1_-FITC (mouse) and IgG_1_-PE (mouse, all Becton Dickinson). In brief, whole blood was stained following the same procedure as the samples [12] using the corresponding isotype control to determine the cut off for the negative and positive populations. Figure 1A shows a representative staining for IFNγ-FITC and CD27-PE as well as their corresponding IgG_1_ isotype controls used to determine the cut off.

Initial gating for each sample set used a forward scatter height (SSC-H) versus a sideward scatter height (SSC-H) plot to isolate small lymphocytes. CD4 versus IFNγ plot was then used to identify MTB-specific CD4 T cell responses. Definition of positive CD4 T cell responses was 3-times background and at least 0.05% IFNγ positive after subtraction of the background. As shown in figure 1B, CD27 positive and CD27 negative CD4 T cells were clearly separate according to their isotype control staining (shown in figure 1A). As negative and positive controls, whole blood was stimulated with PPD diluent and *Staphylococcus aureus* Enterotoxin B (SEB, Sigma), respectively. Figure 1C shows a representative dot plot of a negative and positive control used for each sample. Subjects without a detectable response to SEB (n = 6) or with a high background were excluded (n = 4).

Frozen peripheral blood mononuclear cells (PBMC) from multiple time points of two HIV seroconverters with latent MTB infection were used to study CD27 expression dynamics on MTB-specific CD4 T cells. Latent MTB infection was defined as detectable Region of Difference 1 (RD-1)-specific T cell responses targeting Early Secretory Antigenic Target 6 (ESAT-6) and/or Culture Filtrate Protein 10 (CFP-10). For these samples, flow cytometric studies were performed at the Vaccine Research Center, National Institutes of Health, Bethesda, USA, using a previously described protocol [8]. In brief, after stimulation, PBMC were washed once with PBS and stained with Vivid (Molecular Probes) [13], to exclude dead cells. Subsequently, surface staining was performed with CD4-PE-Cy5.5 (Invitrogen), and CD27 PE-Cy5 (Beckmann Coulter) for 20 min. PBMC were then permeabilized using the Cytofix/Cytoperm kit (BD Biosciences), after which they were stained for CD3 APC-Cy7 and IFNγ FITC (Becton Dickinson), besides other cytokines. PBMC were then washed and fixed with 1% paraformaldehyde and analysis was done using a modified LSR II flow cytometer (BD Immunocytometry Systems). Between 300,000 and 1,000,000 total events were collected from each sample. Electronic compensation was conducted with antibody capture beads (BD Biosciences) stained separately with the individual antibodies while the cut off definition described above were used.

### Statistical analysis

Data analyses were carried out using GraphPad Prism version 4.0 software. Comparisons of two groups were performed using the Mann Whitney test.

## Results

Table 1 summarizes group characteristics and immunological results of the cross-sectional analysis. 59% of TB^−^/HIV^−^ subjects (19/32) and 40% of TB^−^/HIV^+^ subjects (14/35) had detectable PPD-specific CD4 T cell responses (cut off: at least 0.05% IFNγ^+^ CD4 T cells), consistent with previous results that HIV infection is associated with a reduction of PPD-specific CD4 T cell responses [14]. As expected, active TB was associated with detectable PPD-specific CD4 T cell responses; 80% of active TB/HIV^−^ subjects (8/10) and 89% of active TB/HIV^+^ subjects (16/18) had detectable PPD-specific CD4 T cell responses.

**Figure 2:**
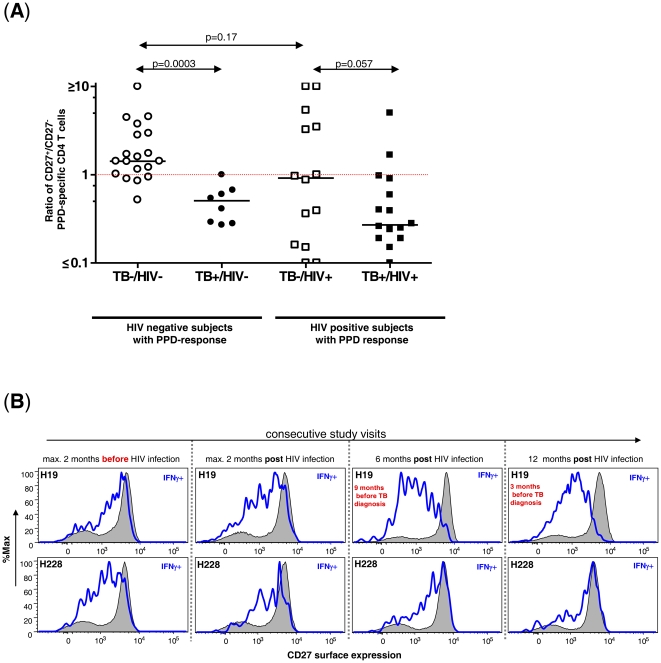
Down regulation of CD27 on PPD-specific CD4 T cells is associated with active Tuberculosis independent of the HIV status and with progression to active TB in a HIV^+^ seroconverter. (A) Ratio of IFNγ^+^ CD4 T cells that are CD27^+^ divided by those that are CD27^−^ is shown for 4 different groups of PPD responders delineated by TB disease state and by HIV serology. CD4^+^ T cells were analyzed for each sample using a whole blood intracellular cytokine assay. (B) Histogram analysis of CD27 expression on IFNγ^+^ PPD-specific CD4 T cells (blue line) and total CD4 T cells (black line, grey) over a 15 months period in two subjects who became HIV infected. Subject H19 (**upper panel**) was diagnosed and treated for active TB 15 months after HIV infection. Subject H228 (**lower panel**) did not develop TB within three years after HIV infection. Longitudinal analysis of CD27 expression for one subject was determined simultaneously by flow cytometry.

Subsequently, the expression of CD27 on PPD-specific CD4 T cells was analysed. In order to compare CD27 expression on PPD-specific CD4 T cells between different PPD responders, the fraction of CD27^+^IFNγ^+^ CD4 T cells was divided through the fraction of CD27^−^IFNγ^+^ CD4 T cells as shown in figure 1B and C. A ratio of >1 defines that a majority of PPD-specific CD4 T cells is characterized by a CD27^+^ phenotype, whereas a ratio <1 defines that a majority of PPD-specific CD4 T cells have a CD27^−^ phenotype. As shown in figure 2A 79% (15/19) of TB^−^/HIV^−^ PPD responders had a ratio >1 with a median ratio of 1.42 (range 0.52–12.29). Only 4 subjects (23%) had a ratio <1. In contrast, 100% of active TB/HIV^−^ subjects (8/8) with a PPD-specific T cell response had a ratio ≤1 (p = 0.0003). The median ratio within the active TB/HIV^−^ group was 0.48 (range 0.28–1.00). These results demonstrate that active TB is associated with down regulation of CD27 on PPD-specific CD4 T cells in the absence of HIV co-infection. In active TB/HIV^+^ subjects with detectable PPD responses, 14/16 (88%) subjects had a ratio of <1. The median ratio was 0.27 (range 0.08–4.99) and was modestly decreased compared to HIV^−^ subjects with active TB (p-value = 0.11). In contrast to the TB^−^/HIV^−^ group, 9/14 TB^−^/HIV^+^ PPD-responders (64%) had a ratio <1 and a median ratio of 0.92 (range 0.0–15.94, Figure 2A). Thus in the presence of HIV infection, CD27 down regulation on PPD-specific CD4 T cells was observed not only in association with active TB, but also within the majority of TB^−^/HIV^+^ subjects. These results are consistent with the hypothesis that chronic HIV infection is frequently associated with low levels of MTB replication in subjects with sub-clinical infection.

We then determined the longitudinal course of CD27 expression on MTB-specific CD4 T cells before and after acquisition of HIV in two IGRA-positive seroconverters with detectable PPD-responses throughout the study [11], [14]. Subject H19, was diagnosed with active TB at 15 month after HIV seroconversion whereas subject H228 stayed TB-asymptomatic despite significantly decreased frequencies of PPD-specific CD4 T cells 12 month post HIV infection [14]. As shown for subject H19 in figure 2B (upper panel), a substantial down regulation of CD27 on MTB-specific T cells was observed as early as 9 month before active TB was diagnosed. The observed down regulation was specific for MTB since total CD4 cells (grey) were not affected. In contrast, expression of CD27 on MTB-specific CD4 T cells was maintained in subject H228 (lower panel) throughout the study period, while remaining TB-asymptomatic. These results suggest that mycobacterial activity in subject H19 preceded clinical disease for several months.

## Discussion

HIV related immunosuppression is associated with increased susceptibility to severe, frequently extrapulmonary tuberculosis [1]. Here we present data suggesting that monitoring PPD-specific T cell responses and their CD27 expression pattern can hold important information about mycobacterial activity in vivo. In subjects with active TB, MTB-specific CD4 T cells dominantly exhibit a more mature CD27^−^ phenotype regardless of the HIV status, whereas in HIV^−^ TB asymptomatic PPD-responders, MTB-specific CD4 T cells exhibit dominantly a less differentiated CD27^+^ phenotype. These data are in line with observations from a low MTB endemic country and suggest that down regulation of CD27 on MTB-specific CD4 T cells correlates with active MTB growth [10]. Likewise, recent studies in a TB mouse model showed that more mature CD27^−^ CD4 T cells are found in the lungs of infected animals and produce significant amounts of IFNγ after stimulation [15].

We have recently shown that MTB-specific CD4 T cell populations are depleted early after HIV infection [8], [14]. However, in many TB asymptomatic subjects with chronic HIV infection these responses are still detectable. The significance of such responses remains largely unknown but might be related to recent exposure, not sufficiently controlled low levels of MTB replication or reactivation that finally leads to clinical TB disease [16]. Interestingly, TB^−^ subjects differed in this marker when stratified by their HIV infection status. MTB-specific CD4 T cells detected in TB^−^/HIV^−^ subjects showed a less differentiated CD27^+^ phenotype, whereas in a majority of TB^−^/HIV^+^ subjects, the phenotype was dominantly CD27^−^. The TB^−^/HIV^+^ subjects were recruited from a HIV/AIDS care and treatment clinic with the majority on ART (table 1) and 9 subjects reported previous episodes of TB disease, suggesting that most of these subjects were in the late, chronic stage of HIV infection. Because re-expression of CD27 on MTB-specific CD4 T cells has been shown to occur very slowly upon TB treatment [10], these data are consistent with the hypothesis that TB^−^/HIV^+^ subjects with CD27^−^ MTB-specific CD4 T cell responses, might have been exposed to subclinical, low levels of MTB replication before commencing ARV treatment. In deed, a follow up after 2 years did not reveal any association of a CD27^−^ phenotype with subsequent reactivating Tuberculosis within HIV^+^ subjects that had started ART. Thus in HIV^+^ subjects on ART the usability of this marker to diagnose active TB might be limited.

Down regulation of CD27 on MTB-specific CD4 T cells preceded the diagnosis of active TB for several months in one individual who continuously mounted MTB-specific responses despite recent HIV infection [14]. In contrast CD27 down regulation was not observed in the subject that stayed TB-asymptomatic throughout the observation period suggesting that HIV infection alone does not explain CD27 down regulation on MTB-specific CD4 T cells. Although it is impossible to draw definite conclusions from only 2 patients, this observation supports the hypothesis that in HIV^+^ subjects MTB growth can start long before clinical disease. However, larger studies are needed to corroborate the predictive value of this assay.

In conclusion we demonstrate that active TB is associated with down regulation of CD27 on MTB-specific CD4 T cells in HIV^−^ subjects from a high TB endemic region. These data suggest that phenotypic analysis of MTB-specific T cells could be used to assess MTB activity in vivo, to assist the diagnosis of TB, and thus to more accurately evaluate the risk of developing active TB.
